# Separation of phenyl acetic acid and 6-aminopenicillanic acid applying aqueous two-phase systems based on copolymers and salts

**DOI:** 10.1038/s41598-021-82476-x

**Published:** 2021-02-10

**Authors:** Farzaneh Ghazizadeh Ahsaie, Gholamreza Pazuki

**Affiliations:** Department of Chemical Engineering, Amirkabir University of Technology (Tehran Polytechnic), Tehran, Iran

**Keywords:** Chemical engineering, Biotechnology

## Abstract

6-Aminopenicillanic acid (6-APA) is used for synthesis of semisynthetic antibiotics. Polymer-salt aqueous two-phase systems (ATPSs) were applied for separation of 6-APA and phenyl acetic acid (PAA), as the products of hydrolyzation reaction of Penicillin G/Penicillin V. The binodal curves of ATPS composed of a copolymer (reverse Pluronic 10R5, Pluronic L35 and PEG-ran-PPG) and a salt (Tri-sodium citrate, tri-potassium citrate, di-potassium phosphate, sodium sulphate and magnesium sulphate) were obtained. The results show that, at a fixed PPG/PEG ratio, block copolymers have larger two-phase region compared with random copolymer. After screening on the partition coefficient of PAA and 6-APA separately, Na_2_SO_4_ was selected for studying the effect of the copolymer structure and the composition of salt and copolymer on partitioning, considering higher selectivity of PAA and 6-APA. 10R5-Na_2_SO_4_ ATPS was selected as the most appropriate system for separation of 6-APA and PAA. This system was used for separation of mixture of 6-APA and PAA. The results show that selectivity was $\approx$ 53 and smaller in a system, containing a mixture of 6-APA and PAA. This observation can be justified by the interaction between 6-APA and PAA. Molecular interaction between these two molecules were investigated by the Flory–Huggins interaction parameter.

## Introduction

Penicillin was firstly discovered by Fleming in 1932. They are widely used in antimicrobial therapy due to their high bacterial activity, low toxicity and good medical efficacy^1^. In order to decelerate the existence of drug-resistance bacteria and ensure the drug influence, now 6-aminopenicillanic acid (6-APA) is used as basic raw material for synthesis of semisynthetic antibiotics^1–4^. 6-APA was discovered in late 1950s^5^. It can be produced by chemical and enzymatic methods. Toxic materials such as pyridine, NOCl and PCl_5_ are used in chemical method which can cause environmental problems. Compare to chemical method, the enzymatic method has several advantages: mild environment, high capacity, low cost and safety^1^. In this method Penicillin G/Penicillin V are hydrolyzed by penicillin acylase to produce 6-APA^4^. The by-product of this synthesis is phenyl acetic acid. Despite the advantage of this method, solubility of Penicillin G, penicillin acylase, 6-APA and PAA cause problems for purification^4^.

In 1896, Beijerinck first observed a phase separation in aqueous solution of starch and gelatin. For the first time, Albertsson applied aqueous two phase system (ATPS), for partitioning of plant cell particles^6–8^. ATPSs can be formed by mixing of two compounds (polymer, salt, ionic liquid, surfactant, to name but a few) in water above a critical concentration^6^. These systems are environment-friendly, non-flammable, scalable, efficient for many purifications and separations and low energy consumption^6,7,9,10^. 6-APA and phenylacetic acid are usually separated from the hydrolysate by precipitation at its pI (isoelectric pH) and n-butyl acetate solvent extraction, respectively. Instead of conventional solvent extraction, aqueous two-phase systems can be an alternative.

The polymer-salt ATPSs are more attractive compared to polymer–polymer ones due to their low viscosity, fast phase separation, low cost and higher selectivity^11^. Several mechanisms for phase separation were suggested. For instance in polymer-salt ATPS, salting out and hydrophobic interactions are considered as dominant mechanisms^12^.

The difference in physicochemical properties of the top and the bottom phases, cause an uneven distribution of biomolecules in ATPS. One of the factors affecting the partitioning of the solute is the phase-forming properties^13^. Copolymers are consisted of two or more monomers. Their properties could be adjusted by manipulating in number of monomers^14^. One of the most widely used copolymers is ethylene glycol (PEG)—propylene glycol (PPG). These copolymers can be in a random (UCON) or a block (normal Pluronic and reverse Pluronic (R-pluronic)) form. Using copolymers as phase forming compounds in ATPSs causes an extreme partitioning, hence selectivity and efficiency increase^15^. Self-assembly of amphiphilic copolymers (block copolymers) in aqueous solutions, enhance the interaction between copolymer and hydrophobic compounds^16,17^. Svensson and et al. indicated that self-assembly of copolymers is not a dominant factor in the partitioning of hydrophilic proteins^18^. Shaker Shiran et al. applied ATPSs based on PEG (Mw = 2000, 6000), PPG400 and Pluronic L35 for curcumin partitioning. Pluronic L35 showed higher potential for partitioning of curcumin^19^.

In this paper, the application of three different PEG-PPG copolymers (random, normal and R-Pluronic) in 6-APA and PAA purification were investigated. Phase behaviors of these copolymers with five different salts were studied. To study the capacity of these ATPSs for separation of both biomolecules, we started by studying the partition behavior of each drug. The effect of salt (anion and cation) and copolymer structure (random, normal and reverse) on binodal curves and partitioning of 6-APA and PAA were investigated. The influence of concentration of salt and copolymers were studied in Na_2_SO_4_-copolymer ATPS. The partition behavior and separation of 6-APA and PAA were investigated for a mixture of them. The effect of temperature on the partitioning of PAA was investigated at 4 different temperatures. In order to study the interaction between 6-APA and PAA, ^1^H-NMR and Flory–Huggins interaction parameter ($\chi_{12}$) were applied. The Flory–Huggins interaction parameter was calculated based on the Hansen solubility parameter.

## Materials and methods

### Materials

**Figure 1:**
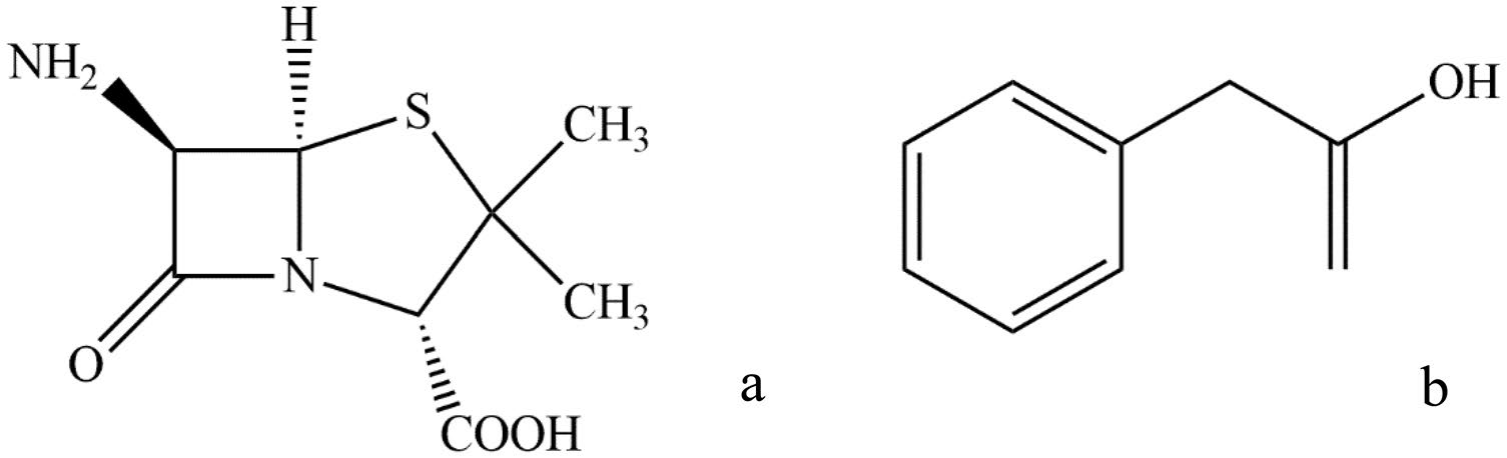
Structure of: (**a**) 6-aminopenicillanic acid (**b**) Phenyl acetic acid.

Pluronic L35 (Mw = 1900), R-Pluronic 10R5 (Mw = 2000) (PO-EO-block polymers with approximately 50% EO), PEG-ran-PPG-ran-PEG (UCON) (Mw = 2500), Tri-sodium citrate, tri-potassium citrate and di-potassium phosphate were purchased from Sigma. Sodium sulphate and magnesium sulphate were acquired from Merck. Phenyl acetic acid and 6-aminopenicillanic acid were acquired from TITRACHEM with mass fraction purity of 98%, prepared in double distilled water. The chemical structures of Phenyl acetic acid and 6-aminopenicillanic acid are represented in Fig. 1.

### Methods

#### Phase diagrams and tie-lines

All phase diagrams were determined at two temperatures (298 and 283 K) and atmospheric pressure through cloud-point titration method described in ref^19^. The experimental data were correlated with Merchuk Eq. (1). The adjustable parameters (A, B, C) were determined by regression of experimental data.

1 $$\left[ {copolymer} \right] = A \times \exp \left[ {\left( {B \times \left[ {Salt} \right]^{0.5} } \right) - \left( {C \times \left[ {salt} \right]^{3} } \right)} \right]$$

The composition of each phase was calculated by Merchuck equation and applying gravimetric method^20^ (Eqs. 2–5).

2 $$\left[ {copolymer} \right]_{Top} = A \times {\text{exp}}\left[ {\left( {B \times \left[ {salt} \right]_{Top}^{0.5} } \right) - \left( {C \times \left[ {salt} \right]_{Top}^{3} } \right)} \right]$$

3 $$\left[ {copolymer} \right]_{Bot} = A \times {\text{exp}}\left[ {\left( {B \times \left[ {salt} \right]_{Bot}^{0.5} } \right) - \left( {C \times \left[ {salt} \right]_{Bot}^{3} } \right)} \right]$$

4 $$\left[ {copolymer} \right]_{Top} = \frac{{\left[ {copolymer} \right]_{F} }}{\alpha } - \frac{1 - \alpha }{\alpha }\left[ {copolymer} \right]_{Bot}$$

5 $$\left[ {salt} \right]_{Top} = \frac{{\left[ {salt} \right]_{F} }}{\alpha } - \frac{1 - \alpha }{\alpha }\left[ {salt} \right]_{Bot}$$

The subscripts F, Top and Bot stand for feed, top and bottom phases, respectively. $\alpha$ is the ratio of top and bottom phase mass. Tie-line length and slope (TLL and STL, respectively) are obtained through Eqs. (6–7).

6 $$TLL = \sqrt {\left( {\left[ {copolymer} \right]_{TOP} - \left[ {copolymer} \right]_{BOT} } \right)^{2} + \left( {\left[ {salt} \right]_{TOP} - \left[ {salt} \right]_{BOT} } \right)^{2} }$$

7 $$STL = \frac{{\left[ {copolymer} \right]_{Top} - \left[ {copolymer} \right]_{Bot} }}{{\left[ {salt} \right]_{Top} - \left[ {salt} \right]_{Bot} }}$$

#### Partitioning of PAA and 6-APA

The salt-copolymer ATPSs were prepared gravimetrically (*u*(*m*) = 10^–4^ g). The mixtures were stirred vigorously and left to reach thermodynamic equilibrium and complete phase separation for at least 24 h at 298 K. Both phases (copolymer-rich and salt -rich phase) were carefully separated and weighed to determine biomolecule concentrations. In this work, the partition coefficient (*K*_*drug*_), the percentage extraction efficiency (*EE%*) and selectivity (Sel) were defined following Eqs. (8–10):

8 $$K_{drug} = \frac{{\left[ {drug} \right]_{Top} }}{{\left[ {drug} \right]_{Bot} }}$$

9 $$EE\% = \frac{{K_{drug} V_{r} }}{{K_{drug} V_{r} + 1}} \times 100$$

10 $$Sel = \frac{{K_{{drug}_{PAA}}}}{{K_{{drug}_{6 - APA}}}}$$

where $\left[ {drug} \right]_{ }$ and $V_{r}$ represent the concentration of drug and the volume ratio between top and bottom phases.

Phenyl acetic acid (PAA) and 6-aminopenicillanic acid (6-APA) were determined by UV Spectroscopy at 254 and 207 nm, respectively, using previously determined calibration curves. In order to eliminate the interference of phase forming components, ternary mixtures at specific concentrations of each component were prepared, using pure water instead of the aqueous solutions of drugs, to be used as blank solution. Three replicates were prepared for each assay, the results were reported as average of triplicated calculated partition coefficient. In order to study the partition coefficient in system contains both 6-APA and PAA, HPLC has been applied. The HPLC procedure is explained in ref.^21^.

#### Effect of temperature on partitioning

To study the effect of temperature on partitioning of PAA, 4 different temperature were selected (4, 15, 25 and 40 °C). According to Eqs. 11–13, thermodynamic parameters of the partitioning of PAA can be determined.

11 $$\Delta G_{m}^{0} = - RT\ln K_{PAA}$$

12 $$\Delta G_{m}^{0} = \Delta H_{m}^{0} - T\Delta S_{m}^{0}$$

13 $$\ln K_{PAA} = - \frac{{\left[ {\Delta H_{m}^{0} /R} \right]}}{T} + \left[ {\Delta S_{m}^{0} /R} \right]$$

Johansson et al. developed a model based on Flory–Huggins to relate the partition coefficient of a solute the enthalpic and entropic contributions^22^. The entropic contribution is given by following equation (Eq. 14):

14 $$\ln K_{drug} = \frac{{M_{N} }}{\rho }\left( {\frac{{n^{T} }}{{V^{T} }} - \frac{{n^{B} }}{{V^{B} }}} \right)$$

where n, V, $M_{N}$, $\rho$, T and B stand for total number of molecules, density number, the density number of ATPS, top phase and bottom phase.

#### Intermolecular interaction

In order to investigate the molecular interaction between PAA and 6-APA, the Hansen solubility parameter has been applied.

The Hansen Solubility parameter has been used to calculate the Flory–Huggins interaction parameter between PAA and 6-APA as an important parameter for interaction estimation^23^. The Hansen Solubility parameter includes three contributions: dispersive ($\delta_{d}$), polar ($\delta_{p} )$, and hydrogen bonding ($\delta_{h}$); as defined in the following:

15 $$\delta_{d} = \frac{{\sum\limits_{i} {F_{{d_{i} }} } }}{{\sum\limits_{i} {V_{i} } }}$$

16 $$\delta_{p} = \frac{{\left( {\sum\limits_{i} {F_{{p_{i} }}^{2} } } \right)^{0.5} }}{{\sum\limits_{i} {V_{i} } }}$$

17 $$\delta_{h} = \frac{{\left( {\sum\limits_{i} {E_{{h_{i} }} } } \right)^{0.5} }}{{\sum\limits_{i} {V_{i} } }}$$

where $F_{d,i}$, $F_{p,i}$ and $F_{h,i}$ are the group contribution of dispersion forces, polar forces and hydrogen bond energy, respectively and $V_{i}$ is the group contribution of structural group i to molar volume. The solubility parameter can be obtained applying Eqs. (15) to (17). The Flory–Huggins interaction parameter can be obtained following Eq. (18):

18 $$\Delta \delta = \sqrt {\left( {\delta_{d,1} - \delta_{d,2} } \right)^{2} + 4\left( {\delta_{p,1} - \delta_{p,2} } \right)^{2} + + \left( {\delta_{h,1} - \delta_{h,2} } \right)^{2} }$$

19 $$\chi_{12} = \left( {\Delta \delta } \right)^{2} V_{1} /RT$$

## Results

### Binodal curves determination

An aqueous mixture of copolymer and salt splits into two phases above certain concentrations of components. The nature of each phase forming can affect the two-phase region. In this work, the effect of the structure of copolymer, temperature and salt anion/cation on the binodal curves were studied. The experimental data were fitted by Merchuk equation (Eq. 1). The Merchuk adjustable parameters are reported in tables S1 and S2.

**Figure 2:**
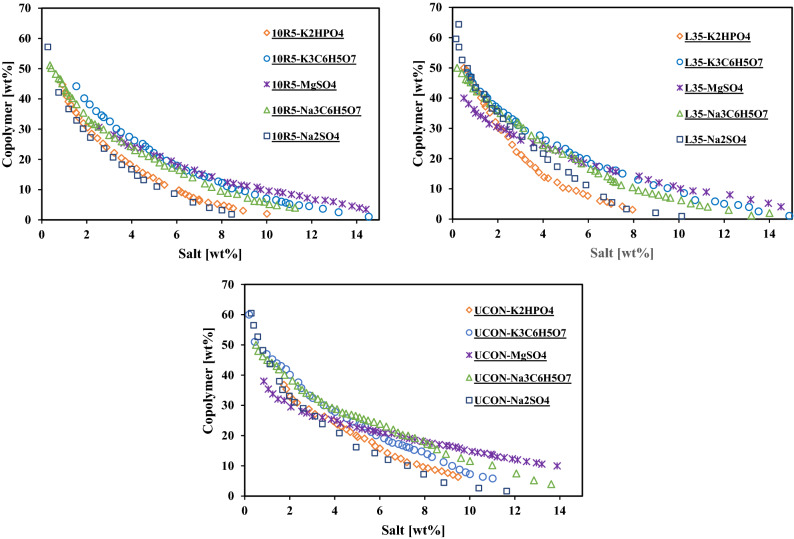
Experimental binodal curves determined for the ATPSs composed of different copolymer + salt at 298 K.

**Figure 3:**
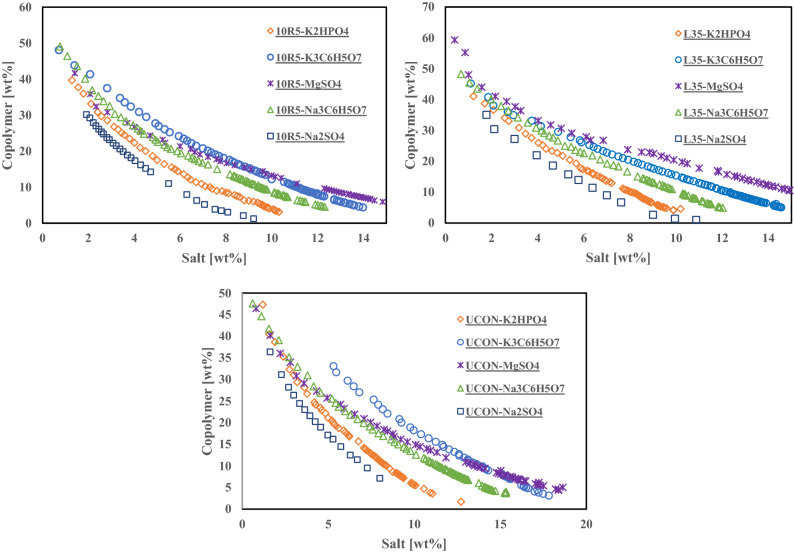
Experimental binodal curves determined for the ATPSs composed of different copolymer + salt at 283 K.

Figures 2 and 3 represent the experimental binodal curves. To eliminate the interference of molecular weight of the different phase forming components on the analysis, the binodal curves are represented in molality scale (figure S1 and S2). The results show that two-phase region expands by increasing the temperature (Figure S3). The solubility and hydrophilicity of the copolymers decrease by increasing the temperature. At higher temperature lower amount of copolymer is needed to form two-phase system. Binodal curves of ATPSs composed of Pluronic L35 and $Na_{2} SO_{4}$/$MgSO_{4}$/$Na_{3} C_{6} H_{5} O_{7}$ at 283.15 and 298.15 K were determined through the characterization of top and bottom phases^24,25^. The obtained binodal curves in this work are in good agreement with reported binodal curves. At a fixed weight fraction of Pluronic 10R5 at 20 wt%, the salting out ability of salts follow the trend:$Na_{2} SO_{4} > K_{2} HPO_{4} > Na_{3} C_{6} H_{5} O_{7} > \approx K_{3} C_{6} H_{5} O_{7} > MgSO_{4}$. Similar trend for these salts are reported in several literatures^26–28^. The salting out ability of $Na_{2} SO_{4}$ and $MgSO_{4}$ are in agreement with literatures^19,24,29,30^.

Assuming complete decomposition of salts, the binodal curves are plotted in the mole fraction of copolymer and ions to investigate the effect of cation and anion (figure S4). In order to study the effect of salt cation on the binodal curve of copolymer/salt ATPS, MgSO_4_/Na_2_SO_4_ and Na_3_C_6_H_5_O_7_/K_3_C_6_H_5_O_7_ were selected. According to figure S4, at higher mole fraction, Na^+^ ion is more able to form two phases compared to Mg^2+^. The salting-out ability of ions can be described based on Hofmeister series and free energy hydration ($\Delta G_{hyd}$). The $\Delta G_{hyd}$ values of Mg^2+^ and Na^+^ are $- 1830\,{\text{kJ}}\,{\text{mol}}^{ - 1}$ and $- 365\,{\text{kJ}}\,{\text{mol}}^{ - 1}$, respectively^29^. More negative $\Delta G_{hyd}$ value for ions leading to more salting-out ability. Although, the Hofmeister series and $\Delta G_{hyd}$ show that Mg^2+^ has more salting-out effect compared to Na^+^ but the obtained results depicted a reverse trend. It should be mentioned that cations with higher valance have more interactions with ether oxygen of PEG monomers in the copolymer structure leading to more salting-in^19^. According to experimental binodal curves of Na_3_C_6_H_5_O_7_ and K_3_C_6_H_5_O_7_ salts, Na^+^ shows similar salting-out effect with K^+^. In order to investigate the effect of anion on the binodal curves, Na_3_C_6_H_5_O_7_/Na_2_SO_4_ and K_3_C_6_H_5_O_7_/K_2_HPO_4_ were chosen. The Hofmeister series for anions follows as^28^: ${\text{HPO}}_{4}^{2 - } < {\text{SO}}_{4}^{2 - } < {\text{C}}_{6} {\text{H}}_{5} {\text{O}}_{7}^{3 - }$. The reported values of $\Delta G_{hyd}$ of ${\text{HPO}}_{4}^{2 - } , {\text{C}}_{6} {\text{H}}_{5} {\text{O}}_{7}^{3 - }$ and ${\text{SO}}_{4}^{2 - }$ are $- 1125, - 2793$ and $- 1080 kJmol^{ - 1}$, respectively. The binodal curves in mole fraction of ions, depicted that both ${\text{SO}}_{4}^{2 - }$ and ${\text{HPO}}_{4}^{2 - }$ has smaller salting out effect than ${\text{C}}_{6} {\text{H}}_{5} {\text{O}}_{7}^{3 - }$. It is in consist with the Hofmeister series and more negative $\Delta G_{hyd}$. This behavior was observed in several literatures^27,31,32^.

Various structures of PEG-PPG copolymers (Pluronic L35, R-Pluronic 10R5 and UCON) contain 50% wt of PEG were investigated. The tendency of phase separation follows as: R-Pluronic 10R5 $\approx {\text{Pluronic L}}35 > {\text{UCON}}$. This trend shows that UCON is more hydrophilic, while the hydrophilicity of R-Pluronic 10R5 and Pluronic L35 is similar. Wu et al. studied the effect of position of PEG and PPG in block copolymer structure on hydrophilic—lipophilic balance (HLB) and relative solubility number (RSN)^33^. RSN and HLB depend on molecular weight, $\frac{{{\text{PEG}}}}{PPG}$ ratio and copolymer structure. They showed that Pluronic copolymers has higher HLB and RSN than corresponding R-Pluronic copolymers. The higher values of HLB and RSN the larger hydrophilicity of copolymers. They also reported that the difference between RSN value is not significant for small copolymers (molecular weight $< 3000$). Reported RSN and HLB values for Pluronic L35, R-Pluronic 10R5 are reported in Table 1. As they have reported the studied block copolymers are similar in hydrophilicity and molecular weight leading to little difference in their binodal curve.

**Table 1:** RSN and HLB values of Pluronic L35 and R-Pluronic 10R5.

Copolymer	RSN	HLB
Pluronic L35	22.3*	18–23**
R-Pluronic 10R5	21.0*	12–18**

*ref.^33^; **ref.^34^.

Several tie-lines were determined for each system at 298 K. The equilibrium concentrations, TLL and STL are reported in table S3-S5. The changes in STL are due to the difference hydrophobicity of the copolymers. In the ATPSs of Pluronic L35, R-Pluronic 10R5 and UCON the absolute value of STL follows: Pluronic L35 $\approx$ R-Pluronic 10R5 $>$ UCON.

### Distribution of biomolecule

In order to evaluate the capacity of these ATPSs in separation of 6APA and PAA, the partition behavior of 6APA and PAA were studied separately. According to the binodal curves at two different temperature, higher temperature was selected due to bigger two-phase region and more hydrophobicity of copolymers at that temperature. The mixture point of 9 and 20 wt% of salt and copolymer, respectively were selected. Since this mixture point in potassium citrate- UCON ATPS is located in one phase region, a mixture point of 13 and 20 wt% of potassium citrate and UCON was studied for this salt. The partition coefficients were reported in Table 2. PAA and 6-APA are considered as hydrophobic and hydrophilic molecules, respectively, due to their octanol–water partition coefficients $\log K_{OW,PAA} = 1.43$^35^ and $\log K_{OW,6 - APA} = - 0.8$^36^. As it is obvious, PAA has more affinity to the top phase (hydrophobic phase). In all studied ATPSs, except in K_2_HPO_4_ and K_3_C_6_H_5_O_7_ based one, the hydrophilic molecule (6-APA) is more concentrated in the bottom phase as Vobecka and co-workers reported that partition coefficient of 6-APA was greater than one in K_2_HPO_4_-PEG ATPS^37^. Since K_2_HPO_4_ and K_3_C_6_H_5_O_7_ are two basic salts, pH value of the ATPSs based on them is basic. 6-APA has no net charge at its Isoelectric pH (pI = 3.9)^38^, but it is negatively charged in K_2_HPO_4_ and K_3_C_6_H_5_O_7_ (pH > pI). 6-APA shows more affinity to the top phase in K_2_HPO_4_ and K_3_C_6_H_5_O_7_ based ATPSs, despite the more hydrophobicity of top phase. Haghtalab et al. observed similar behavior for the partitioning of α-amylase in PEG—K_2_HPO_4_ ATPS. They reported that increasing pH value, causes an enhancement in the partition coefficient of α-amylase (pH > pI = 5.4)^39^. Several authors have reported that negatively charged molecules show more affinity to the (PEG/ copolymer PEG-PPG)-rich phase since PEG polymer behaves as positively charged molecule^40–42^. Vobecka et al. studied the partition coefficients of PAA and 6-APA in five different ATPSs composed of PEG (mw = 400 and 4000), dextran, citrate and phosphate salts. According to their results, PAA showed more affinity to the more hydrophobic phase (PEG-rich phase).The highest partition coefficients were reported in ATPS based on PEG400 and phosphate salts ($\approx 25$)^37^. Xue-jun et al. applied PEG-dextran ATPSs to investigate the partition behavior of PAA and 6-APA. Their results showed that both PAA and 6-APA distributed evenly between two phases^43^. In all studied systems, Na_2_SO_4_-ATPSs have the highest partition coefficient and the lowest water ratio between top and bottom phases. Water ratio values are reported in table S6. All the reported values are lower than one (less water in the top phase) hence the top phase is more hydrophobic.

**Table 2:** Partition coefficient (K), extraction efficiency (EE%) and selectivity (Sel.) of 6-APA and PAA in systems based in copolymers + salts + water.

Copolymer	Salt	$K_{6 - APA} \pm \sigma$	$K_{PAA} \pm \sigma$	$EE_{6 - APA} \pm \sigma$	$EE_{PAA} \pm \sigma$	$K_{PAA} /K_{6 - APA}$
Pluronic 10R5	K_3_C_6_H_5_O_7_	1.04 ± 0.07	4.4 ± 0.20	66.62 ± 1.68	75.95 ± 0.95	4.60
	K_2_HPO_4_	1.49 ± 0.01	5.99 ± 0.40	49.85 ± 0.17	80.20 ± 1.27	4.05
	Na_3_C_6_H_5_O_7_	0.2 ± 0.10	2.26 ± 0.38	89.43 ± 5.67	54.60 ± 8.29	10.42
	MgSO_4_	0.52 ± 0.06	4.36 ± 0.12	89.09 ± 1.23	60.88 ± 0.66	8.47
	Na_2_SO_4_	0.78 ± 0.08	19.95 ± 0.44	60.89 ± 2.51	94.14 ± 0.14	24.92
Pluronic L35	K_3_C_6_H_5_O_7_	1.01 ± 0.08	6.24 ± 0.40	66.62 ± 1.76	76.89 ± 1.21	6.64
	K_2_HPO_4_	1.7 ± 0.01	4.8 ± 0.08	49.26 ± 0.07	74.27 ± 0.33	2.80
	Na_3_C_6_H_5_O_7_	0.47 ± 0.20	1.79 ± 0.38	82.21 ± 8.05	46.24 ± 5.20	3.85
	MgSO_4_	0.44 ± 0.00	5.90 ± 0.90	90.61 ± 0.01	55.82 ± 3.01	12.30
	Na_2_SO_4_	0.52 ± 0.08	49.00 ± 8.60	72.98 ± 0.70	97.06 ± 0.49	91.52
UCON	K_3_C_6_H_5_O_7_	3.30 ± 0.01	4.97 ± 0.13	35.39 ± 3.79	73.45 ± 1.57	1.49
	K_2_HPO_4_	2.12 ± 0.19	6.14 ± 0.28	45.66 ± 2.40	77.05 ± 1.28	2.81
	Na_3_C_6_H_5_O_7_	0.40 ± 0.10	1.20 ± 0.02	82.47 ± 0.00	47.05 ± 11.47	4.70
	MgSO_4_	0.58 ± 0.01	6.80 ± 0.20	89.91 ± 0.16	56.80 ± 2.09	11.76
	Na_2_SO_4_	0.70 ± 0.01	16.6 ± 0.50	62.73 ± 0.50	93.81 ± 2.55	30.07

After the investigation of the effect of salt, Na_2_SO_4_ was selected for further study, considering higher partition coefficient for PAA and higher $\frac{{{\varvec{K}}_{{{\varvec{PAA}}}} }}{{{\varvec{K}}_{{6 - {\varvec{APA}}}} }}\user2{ }$ ratio. 9 mixture points (Table 3) were chosen to study the effect of salt and copolymer weight fraction. The results are shown in Table 3. At a constant weight fraction of copolymer (salt), an enhancement in salt (copolymer) weight fraction causes an increase in TLL, hence the weight fraction of copolymer and salt in top and bottom phases increases, respectively. So, PAA can have interaction with more copolymer molecules and leads to higher partition coefficient of PAA (TLL are reported in tables S3-S4). The effect of TLLs on the partition coefficient is represented in figure S5. As Sousa et al. reported, the effect of tie-line length on the partitioning of biomolecules is depend on the biomolecule’s nature^44^. In all studied systems increasing in tie-line causes an increase in the partition coefficient of PAA. While partition coefficient of 6-APA has no significant dependent on tie-line length.

**Table 3:** Weight percentage composition of each mixture point tested ($W_{t}$%), partition coefficient (K), extraction efficiency (EE%) and selectivity (Sel.) for systems based in copolymers and Na_2_SO_4_.

[Salt copolymer] $W_{t}$%	Pluronic 10R5			Pluronic L35			UCON		
	$K_{PAA} \pm \sigma$	$K_{6 - APA} \pm \sigma$	**Sel**	$K_{PAA} \pm \sigma$	$K_{6 - APA} \pm \sigma$	**Sel**	$K_{PAA} \pm \sigma$	$K_{6 - APA} \pm \sigma$	$K_{PAA} /K_{6 - APA}$
[9-30]	76.14 ± 1.30	0.54 ± 0.06	141	66.67 ± 1.23	0.42 ± 0.02	158	41.94 ± 0.34	0.69 ± 0.06	61
[9 25]	26.15 ± 0.12	0.25 ± 0.02	104	40.87 ± 0.62	0.47 ± 0.03	87	27.42 ± 1.11	0.63 ± 0.03	44
[9 20]	19.54 ± 0.65	0.78 ± 0.08	25	38.83 ± 0.85	0.43 ± 0.02	91	21.00 ± 1.63	0.71 ± 0.02	30
[9 15]	18.92 ± 1.27	0.15 ± 0.02	123	11.45 ± 0.22	0.25 ± 0.09	46	13.05 ± 0.41	0.54 ± 0.02	24
[9–10]	11.87 ± 1.03	0.48 ± 0.06	25	11.13 ± 0.10	0.54 ± 0.04	21	7.25 ± 0.19	0.75 ± 0.09	10
[5 20]	7.81 ± 0.24	0.91 ± 0.06	9	8.30 ± 0.26	0.50 ± 0.07	17	4.02 ± 0.6	0.84 ± 0.13	5
[7 20]	16.73 ± 5.57	0.58 ± 0.11	29	20.20 ± 1.18	0.54 ± 0.00	37	11.35 ± 0.58	0.43 ± 0.05	26
[11 20]	41.44 ± 1.42	0.33 ± 0.01	124	39.67 ± 0.94	0.64 ± 0.01	62	27.07 ± 2.32	0.39 ± 0.10	69
[13 20]	79.61 ± 0.81	0.40 ± 0.09	199	77.47 ± 2.58	0.48 ± 0.07	160	59.01 ± 1.95	1.22 ± 0.13	48

	$EE_{PAA} \pm \sigma$	$EE_{6 - APA} \pm \sigma$		$EE_{PAA} \pm \sigma$	$EE_{6 - APA} \pm \sigma$		$EE_{PAA} \pm \sigma$	$EE_{6 - APA} \pm \sigma$	
[9–30]	98.94 ± 0.11	61.94 ± 2.50		97.09 ± 0.08	82.65 ± 0.56		98.44 ± 0.01	57.65 ± 1.64	
[9 25]	96.61 ± 0.08	78.61 ± 1.47		97.79 ± 0.18	66.42 ± 1.19		96.48 ± 0.13	61.37 ± 1.23	
[9 20]	94.14 ± 0.14	60.89 ± 2.51		97.06 ± 0.49	72.98 ± 0.70		93.81 ± 2.55	62.73 ± 0.50	
[9 15]	91.20 ± 0.55	92.23 ± 0.96		86.71 ± 0.55	87.86 ± 4.03		89.73 ± 0.29	73.56 ± 0.69	
[9–10]	75.43 ± 1.55	88.99 ± 1.55		77.98 ± 0.15	85.43 ± 0.94		72.50 ± 0.53	78.70 ± 2.05	
[5 20]	92.13 ± 0.23	42.35 ± 1.64		94.32 ± 0.17	50.24 ± 3.38		91.54 ± 1.17	30.58 ± 3.50	
[7 20]	94.19 ± 0.15	64.14 ± 4.20		95.68 ± 0.15	62.59 ± 0.20		91.89 ± 0.37	70.01 ± 2.40	
[11 20]	96.52 ± 0.12	81.75 ± 0.42		96.09 ± 0.12	71.49 ± 0.39		94.74 ± 0.43	79.35 ± 4.04	
[13 20]	96.95 ± 0.04	86.38 ± 2.55		97.21 ± 0.09	82.20 ± 2.29		97.79 ± 0.07	52.42 ± 2.59	

Although Pluronic L35 has higher value of HLB and RSN which lead to more hydrophilicity but the hydrophobic molecule (PAA) showed more tendency to the top phase in ATPS based on Pluronic L35 compared with Pluronic 10R5. This observation can be explained by the higher ability of Pluronic L35 in micellization than Pluronic 10R5. Self-aggregation behavior in reverse and normal Pluronic is different^45^. Normal Pluronic forms star-like micelles, while R-Pluronic can form flower-like or random network micelles^46,47^. Micellization is a more favorable process in normal Pluronic solution compared with reverse one^46^. Aggregation number and CMC in R-Pluronic solution is smaller and higher than normal Pluronic^48^, respectively. The obtained results show that R-Pluronic has lower ability in increasing the solubility of hydrophobic molecules than normal Pluronic. Similar results were reported for solubility and partitioning of toluene and 1,2-dichloroethane ( hydrophobic molecules) in normal and R-Pluronic solutions^49,50^.

According to the reported partition coefficient for PAA and 6-APA in Table 3, maximum of ${\text{K}}_{{{\text{PAA}}}} /{\text{K}}_{{6 - {\text{APA}}}}$ ratio is in Pluronic 10R5- Na2SO4. To study the capacity of these ATPS for simultaneous partitioning and separation of PAA and 6-APA in mixture, Pluronic 10R5 – Na2SO4 ATPS was selected considering higher ${\text{K}}_{{{\text{PAA}}}} /{\text{K}}_{{6 - {\text{APA}}}}$ ratio, ${\text{EE}}_{{{\text{PAA}}}}$ and ${\text{EE}}_{{6 - {\text{APA}}}}$. The obtained results are reported in Table 4. The results show that selectivity of PAA and 6-APAA in this ATPS is approximately 53. Reduction in selectivity shows that there is an interaction between 6-APA and PAA.

**Table 4:** Weight percentage composition ($W_{t}$%), partition coefficient (K), extraction efficiency (EE%) and selectivity (Sel.) for a mixture of PAA and 6-APA in 10R5- Na_2_SO_4_.

[salt copolymer] $W_{t}$%	$K_{PAA}$	$K_{6 - APA}$	Sel
10R5 – Na_2_SO_4_ [RS 11 20]	39.62	0.74	53.54

### Effect of temperature on partition coefficient

Partition coefficients of PAA at four different temperatures are reported in Table 4. The results show that an increase in temperature causes an enhancement in ${\text{K}}_{{{\text{PAA}}}}$. The enhancement of hydrophobicity of PEG-PPG copolymers due to increasing the temperature leads to more hydrophobic interaction between copolymer and PAA. Standard molar enthalpy and entropy are calculated through Eq. 13. Partitioning of PAA is a spontaneous and endothermic process since $\Delta H_{m}^{0}$ and $\Delta G_{m}^{0}$ are positive and negative, respectively.

$\Delta S_{m}^{0}$ shows the decrease or increase in the number of distributing of the components in the systems. Change in the entropy can be due to the transferring of the drug ($\Delta S_{m,drug}^{0}$) and other components in the systems ($\Delta S_{m,comp}^{0}$).

20 $$\Delta S_{m}^{0} = \Delta S_{m,drug}^{0} + \Delta S_{m,comp}^{0}$$

According to the Eq. 14, the entropy PAA migration from the phase with higher number density (bottom phase with higher water content) to the other phase is unfavorable entropically ($\Delta S_{m,drug}^{0} < 0$)^51^. The interaction between PAA and Pluronic 10R5, release the water molecules from the ether groups in the Pluronic 10R5 and move them to the higher-number-density phase (bottom phase)^51,52^. The value of $T\Delta S_{m}^{0}$ at 298.15 K is reported in Table 5. Since $T\Delta S_{m}^{0} > \Delta H_{m}^{0}$ partitioning of PAA is entropic- driven.

**Table 5:** Molar standard thermodynamic of partitioning of PAA.

Temperature (°C)	${\text{K}}_{{{\text{PAA}}}}$	$\Delta H_{m}^{0} \left( {{\text{kJ}}\,{\text{mol}}^{ - 1} } \right)$	$\Delta S_{m}^{0} \,{\text{kJ}}\,{\text{mol}}^{ - 1} \,{\text{K}}^{ - 1}$	$T\Delta S_{m}^{0}$
4	8.77	28.46	0.12	35.76
15	12.86			
25	18.92			
40	37.21			

### Intermolecular interaction

The Flory–Huggins interaction parameter ($\chi_{ij}$) can be used to study the compatibility between two components. Table 6 represents the values of $\chi_{12}$. The lower value of $\chi_{12}$, the higher compatibility and more favorable interaction for PAA-X pair (X: copolymer, water, 6-APA)^53,54^. The compatibility with PAA follows the order: 6-APA $>$ copolymer $>$ water. It can be concluded that $K_{PAA} > 1$ and higher concentration of PAA in copolymer-rich phase is due to the higher affinity of PAA to copolymer. The smaller selectivity for separation of a concentrated mixture of PAA and 6-APA can be justified by the affinity of 6-APA with PAA.

**Table 6:** Hansen solubility parameters and $\chi_{12}$ values for all compounds at 298 K.

	$\delta_{d}$	$\delta_{p}$	$\delta_{h}$	$\delta_{t}$	$\chi_{12}$
PAA	21.23	4.45	10.12	23.94	-
6-APA	27.09	10.74	16.14	33.31	0.21
Copolymer	17.22	1.5	9.5	19.70	0.74
Water	15.5	16.0	42.3	47.8	7.7

## Conclusion

In this study, the phase behavior of three different structures of copolymers and five salts were studied. The respective binodal curves and tie lines were determined. The results show that block copolymers (Pluronic L35 and R-Pluronic 10R5) have more ability for phase separation. These systems were applied for partitioning of PAA and 6-APA. The hydrophobic interaction and salting- out ability of salts have significant effects on partition coefficient of both PAA and 6-APA. It was concluded that the most hydrophobic solute has partitioned preferentially towards the most hydrophobic phase and the most hydrophilic solute for the most hydrophilic phase. According to the values of selectivity, these ATPSs shows high potential for separation of 6-APA and PAA.

## Supplementary Information

Supplementary Information.
