# Supplementary material for: Separation of phenyl acetic acid and 6-aminopenicillanic acid applying aqueous two-phase systems based on copolymers and salts
Source: Sci Rep. 2021 Feb 10;11:3489. doi: 10.1038/s41598-021-82476-x (PMC7875977; doi:10.1038/s41598-021-82476-x)
Supplement: Supplementary file 1 — Supplementary Information. [file 41598_2021_82476_MOESM1_ESM.pdf]

Separation of Phenyl Acetic Acid and 6-aminopenicillanic Acid Applying Aqueous Two-Phase Systems based on Copolymers and Salt

Farzaneh Ghazizadeh Ahsaie, Gholamreza Pazuki*

[1]Department of Chemical Engineering, Amirkabir University of Technology (Tehran Polytechnic), Tehran, Iran

* Corresponding author: Tel: +98-021-64543159. Fax: +98-021-66405847. E-mail address: ghpazuki@aut.ac.ir (G.R. Pazuki)

Table S1. Merchuk parameters and correlation coefficient (R^2) obtained by fitting Eq. 1. (298 K)

Salt	**Copolymer**	$A \pm \sigma$	$B \pm \sigma$	$10^3(C \pm \sigma)$	R^2
**$MgSO_4$**	Pluronic L35	52.1 ± 6.7	-0.382 ± 0.085	0.34 ± 0.2	0.997
	UCON	45.1 ± 6.8	-0.279 ± 0.090	0.20 ± 0.11	0.998
	Pluronic 10R5	64.4 ± 5.4	-0.472 ± 0.060	0.39 ± 0.14	0.993
**$Na_3C_6H_5O_7$**	Pluronic L35	61.1 ± 8.2	-0.393 ± 0.122	1.22 ± 0.83	0.991
	UCON	66.6± 5.3	-0.393 ± 0.066	0.50 ± 0.18	0.998
	Pluronic 10R5	71.4 ± 6.4	-0.520 ± 0.079	0.89 ± 0.09	0.996
**Na_2SO_4**	Pluronic L35	77.1±3.5	-0.541±0.049	0.32±0.01	0.995
	UCON	86.0±13.8	-0.655±0.193	1.35±0.11	0.999
	Pluronic 10R5	83.6±9.0	-0.740±0.126	2.5±0.17	0.998
**K_2HPO_4**	Pluronic L35	89.9± 14.3	-0.775 ± 0.145	2.64 ± 0.17	0.988
	UCON	68.9 ± 8.0	-0.499 ± 0.106	1.10 ± 0.59	0.998
	Pluronic 10R5	88.2 ± 13.2	-0.740 ± 0.123	1.78 ± 0.92	0.994
**$K_3C_6H_5O_7$**	Pluronic L35	71.9 ± 3.8	-0.481 ± 0.038	0.60 ±0.12	0.999
	UCON	75.6 ± 9.1	-0.458 ± 0.118	0.80 ± 0.64	0.984
	Pluronic 10R5	99.0 ± 8.1	-0.627 ± 0.054	0.66 ± 0.19	0.998

Table S2. Merchuk parameters and correlation coefficient (R^2) obtained by fitting Eq. 1. (283 K)

Salt	**Copolymer**	$A \pm \sigma$	$B \pm \sigma$	$10^3(C \pm \sigma)$	R^2
**$MgSO_4$**	Pluronic L35	73.7±2.2	-0.380±0.018	0.70 ±0.02	0.994
	UCON	69.1±1.2	-0.438±0.009	0.13±0.01	0.999
	Pluronic 10R5	72.4±1.7	-0.487±0.0130	0.18±0.01	0.998
**$Na_3C_6H_5O_7$**	Pluronic L35	66.5±1.2	-0.368±0.011	0.72±0.02	0.998
	UCON	68.1±1.3	-0.408±0.012	0.37±0.01	0.997
	Pluronic 10R5	74.4±1.6	-0.484±0.014	0.6±0.03	0.997
**Na_2SO_4**	Pluronic L35	64.1±10.1	-468±0.103	2.19±0.44	0.997
	UCON	81.7 ± 2.7	-0.634 ± 0.021	1.2 ± 0.00	1.000
	Pluronic 10R5	74.4±2.9	-0.642±0.025	2.61±0.10	1.000
**K_2HPO_4**	Pluronic L35	65.6±2.0	-0.405±0.018	1.50±0.05	0.999
	UCON	85.6±2.3	-0.571±0.016	0.86±0.04	0.998
	Pluronic 10R5	82.2±2.5	-0.622±0.019	1.10±0.05	0.999
**$K_3C_6H_5O_7$**	Pluronic L35	62.9±1.0	-0.328±0.008	0.38±0.00	0.999
	UCON	84.8±7.6	-0.402±0.036	0.20± 0.02	0.998
	Pluronic 10R5	69.4±1.7	-0.385±0.014	0.48±0.02	0.998

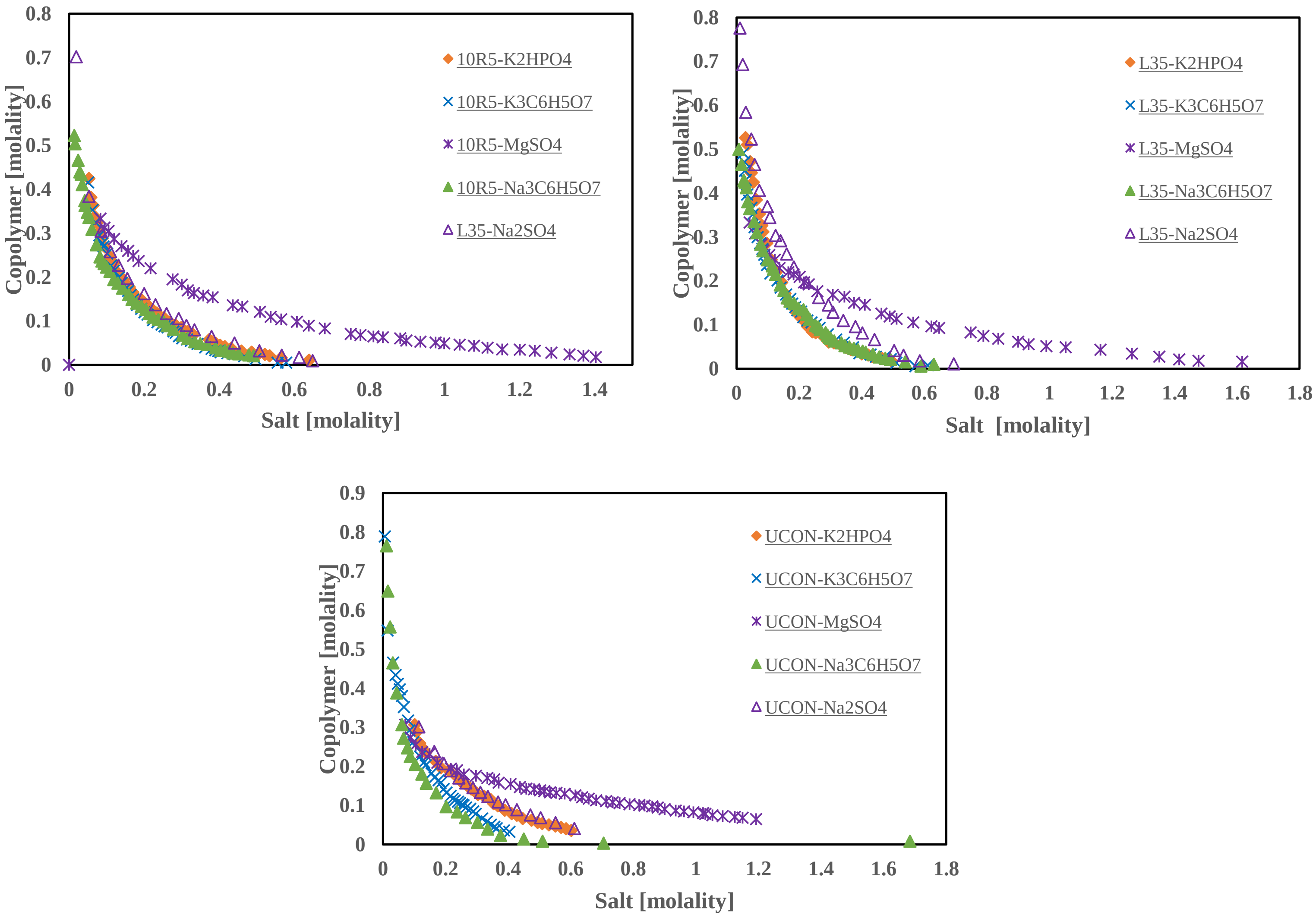

Figure S1. Experimental binodal curves determined for the ATPSs composed of different copolymers + salts in molality at 298 K

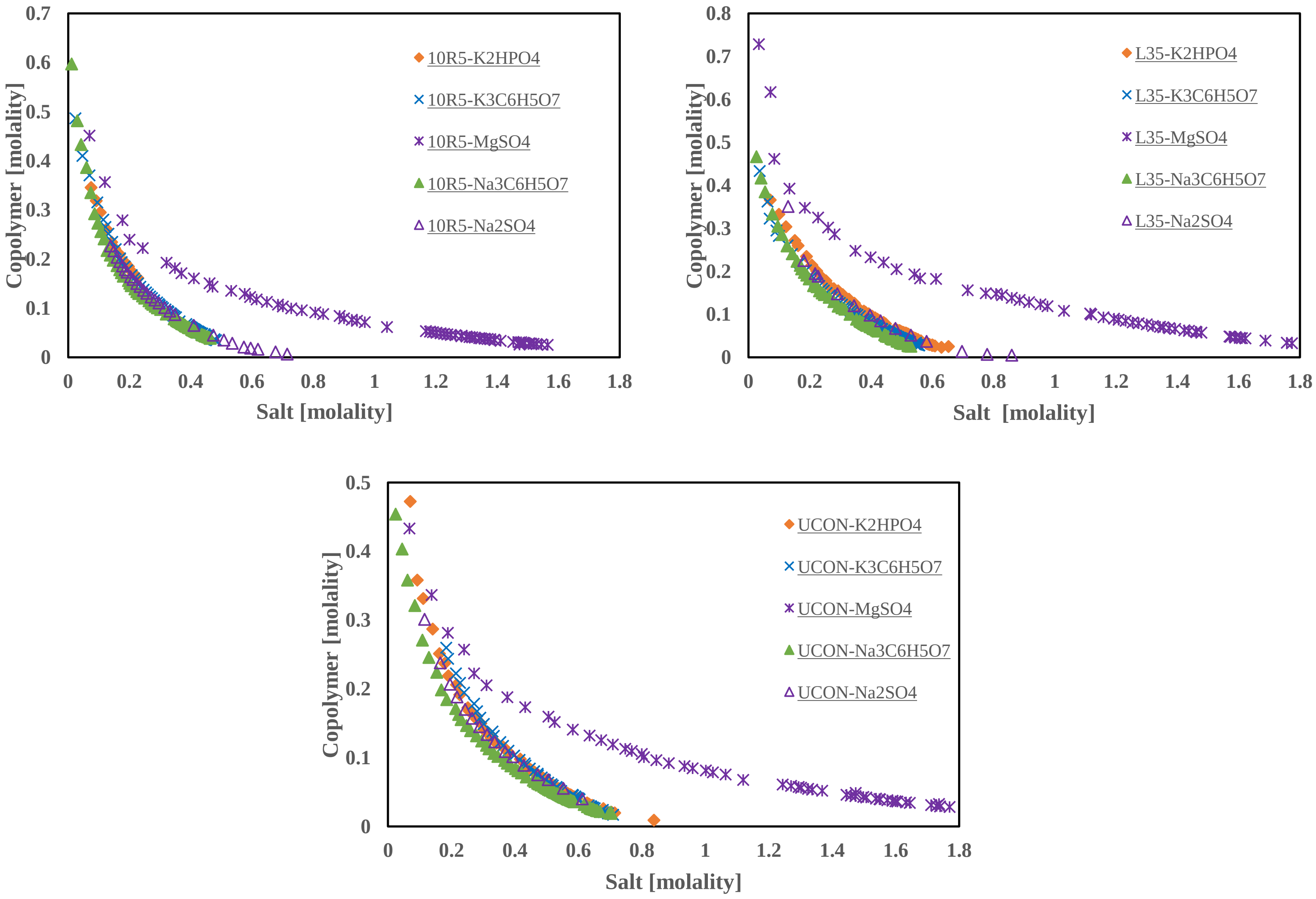

Figure S2. Experimental binodal curves determined for the ATPSs composed of different copolymers + salts in molality at 283 K

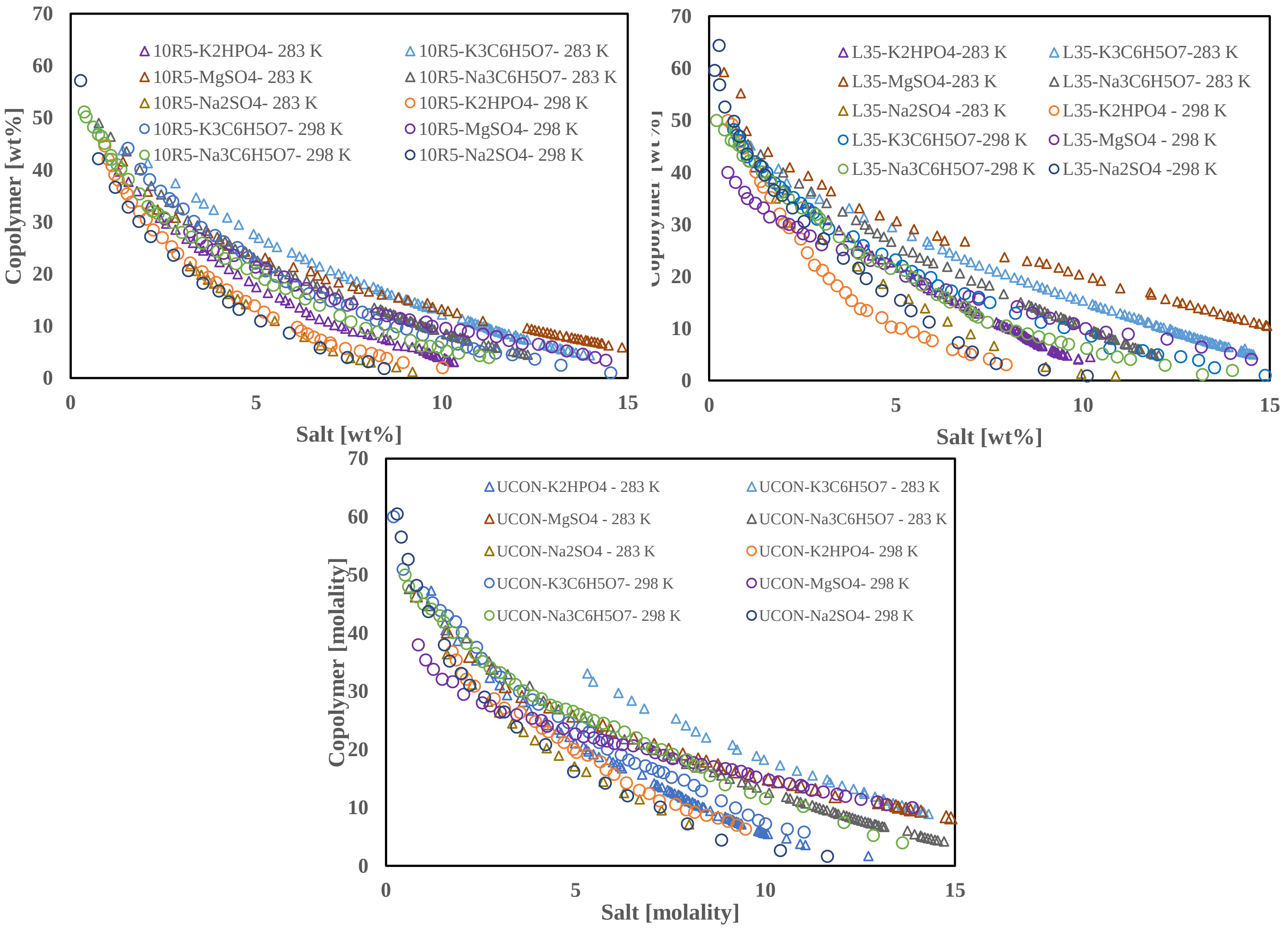

Figure S3. Binodal curves at 298.15 K and 283.15 K

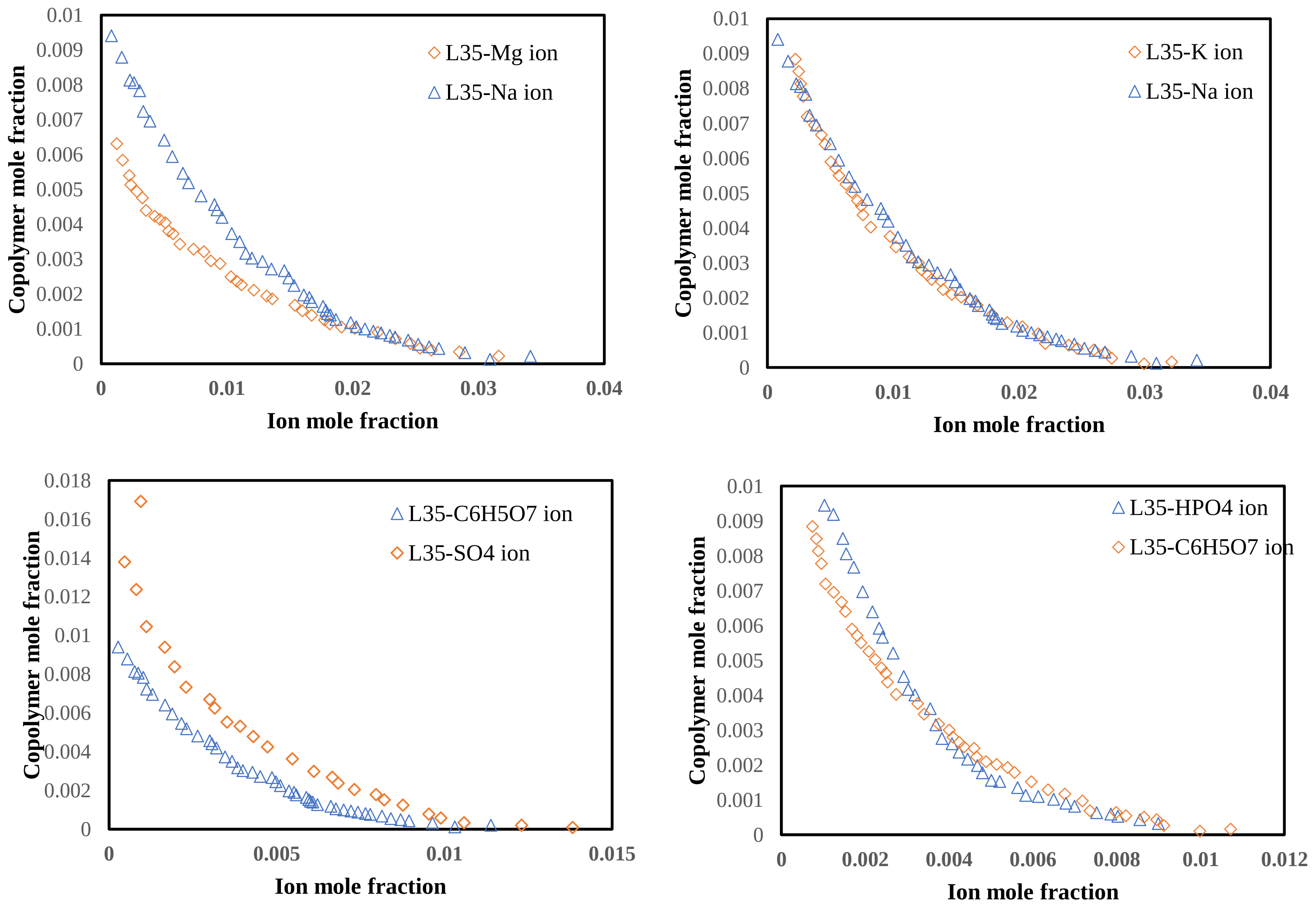

L35-Mg ion
L35-Na ion
Copolymer mole fraction
Ion mole fraction
0
0.001
0.002
0.003
0.004
0.005
0.006
0.007
0.008
0.009
0.01
0
0.01
0.02
0.03
0.04
L35-K ion
L35-Na ion
Copolymer mole fraction
Ion mole fraction
0
0.001
0.002
0.003
0.004
0.005
0.006
0.007
0.008
0.009
0.01
0
0.01
0.02
0.03
0.04
L35-C6H5O7 ion
L35-SO4 ion
Copolymer mole fraction
Ion mole fraction
0
0.002
0.004
0.006
0.008
0.01
0.012
0.014
0.016
0.018
0
0.005
0.01
0.015
L35-HPO4 ion
L35-C6H5O7 ion
Copolymer mole fraction
Ion mole fraction
0
0.001
0.002
0.003
0.004
0.005
0.006
0.007
0.008
0.009
0.01
0
0.002
0.004
0.006
0.008
0.01
0.012

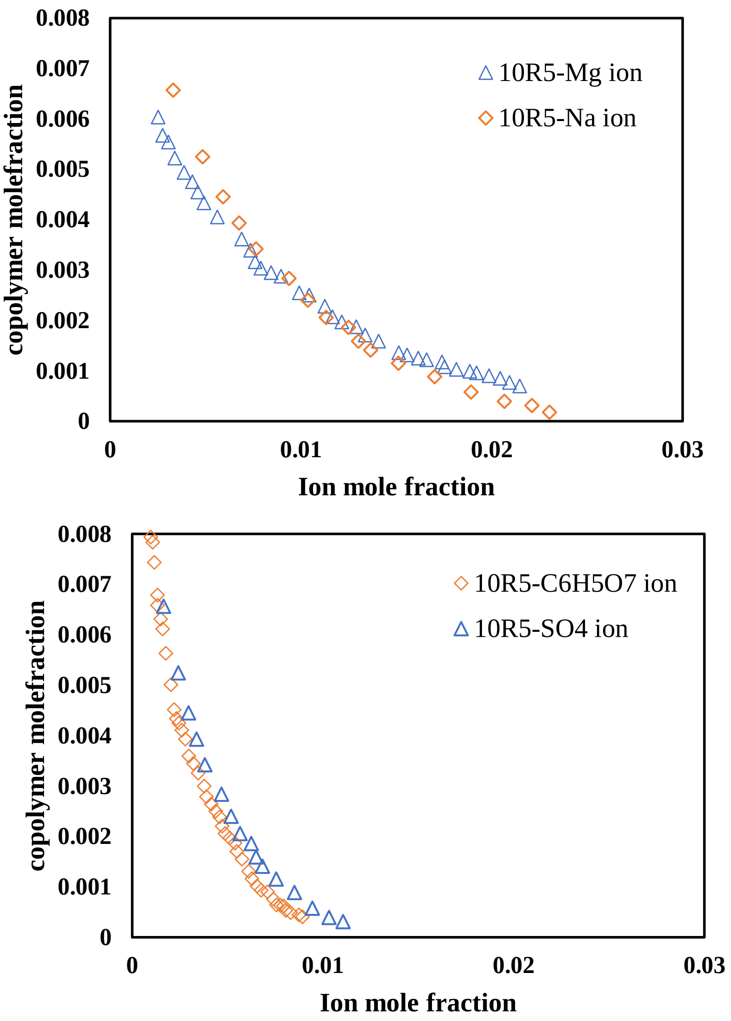

0.008
0.007
0.006
0.005
0.004
0.003
0.002
0.001
0
copolymer molefraction
0
0.01
0.02
0.03
Ion mole fraction
10R5-Mg ion
10R5-Na ion
10R5-K ion
10R5-Na ion
10R5-C6H5O7 ion
10R5-SO4 ion
10R5-HPO4 ion
10R5-C6H5O7 ion

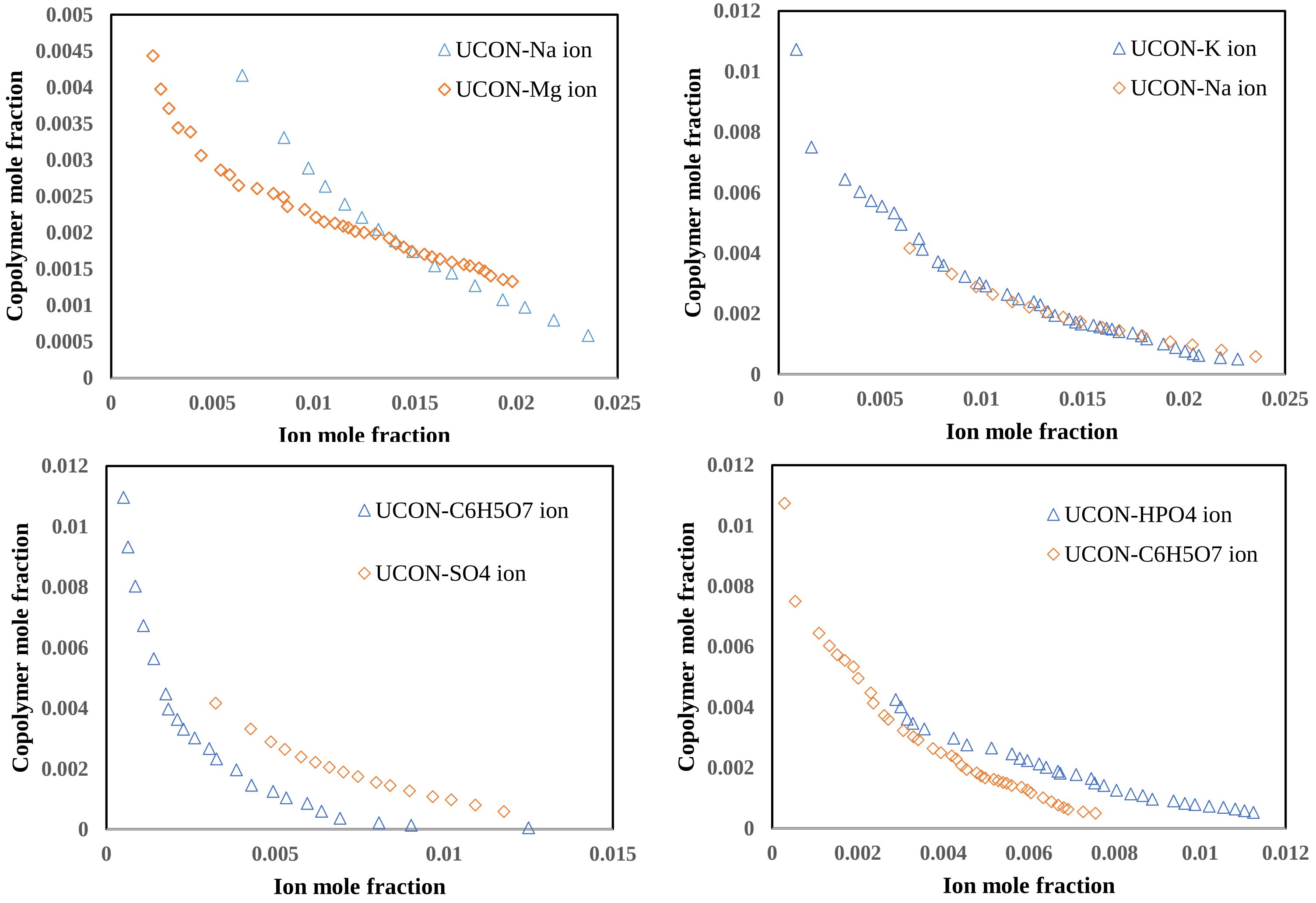

Figure S4. Effect of ions on the binodal curves in ATPSs based on Pluronic L35, Pluronic 10R5 and UCON

Table S3. Phase composition, tie-line data of the ATPS formed by R-Pluronic 10R5(1) + salt (2)

Salt	overall		Top phase		Bottom phase		TLL	STL
	$100w_1$	$100w_2$	$100w_1$	$100w_2$	$100w_1$	$100w_2$		
$MgSO_4$	19.17	9.08	29.80	2.62	0.61	21.66	35.24	-1.56
	19.69	10.99	32.70	2.04	0.02	24.52	39.67	-1.45
	20.48	12.95	35.62	1.57	0.00	44.57	44.57	-1.33
$Na_3C_6H_5O_7$	19.28	8.92	34.96	1.86	0.06	17.57	38.27	-2.22
	19.57	11.12	39.66	1.27	0.00	20.71	44.16	-2.04
	19.51	13.10	44.59	0.90	0.00	22.98	49.75	-2.02
Na_2SO_4	19.98	10.98	48.91	0.53	0.00	18.20	52.01	-2.77
	20.03	9.01	46.69	0.72	0.00	15.74	47.15	-2.98
	24.97	9.00	47.82	0.57	0.00	18.20	50.97	-2.71
	14.97	8.98	42.21	0.85	0.02	13.44	44.03	-3.35
	20.01	7.01	40.94	0.93	0.03	12.80	42.60	-3.34
	19.96	4.99	31.45	1.71	0.33	10.68	32.38	-3.47
	19.19	12.48	51.68	0.43	0.00	19.75	55.17	-2.67
	9.92	8.92	34.30	1.44	0.08	11.97	35.80	-3.24
	29.94	8.98	52.72	0.39	0.00	20.36	56.37	-2.64
K_2HPO_4	20.20	8.95	40.58	1.095	0.00	16.74	43.49	-2.59
	20.26	10.99	45.70	0.79	0.00	19.12	49.24	-2.49
	20.09	12.89	47.40	0.70	0.00	21.86	51.88	-2.25
$K_3C_6H_5O_7$	20.80	8.91	34.84	2.82	0.14	18.10	37.59	-2.25
	20.51	10.95	40.04	2.11	0.03	20.22	43.92	-2.21
	19.85	12.93	41.59	1.95	0.00	22.96	46.59	-1.98

Table S4. Phase composition, tie-line data of the ATPS formed by Pluronic L35(1) + salt (2)

Salt	overall		Top phase		Bottom phase		TLL	STL
	$100w_1$	$100w_2$	$100w_1$	$100w_2$	$100w_1$	$100w_2$		
$MgSO_4$	20.85	8.97	30.03	2.06	0.05	24.62	37.52	-1.33
	20.07	11.11	32.94	1.43	0.02	26.19	41.19	-1.33
	20.76	12.90	37.37	0.76	0.01	28.07	44.57	-1.37
$Na_3C_6H_5O_7$	19.39	12.46	43.38	0.76	0.00	21.92	48.27	-2.05
	21.16	9.19	37.49	1.52	0.00	19.14	41.39	-2.13
	20.42	11.02	41.49	0.97	0.00	20.77	45.98	-2.09
Na_2SO_4	25.01	9.00	50.62	0.60	0.00	17.19	53.27	-3.05
	20.02	7.00	38.21	1.62	0.01	12.93	38.84	-3.49
	19.40	10.97	52.08	0.52	0.00	17.44	54.76	-3.08
	20.02	9.01	47.72	0.78	0.00	14.93	49.78	-3.37
	14.97	8.97	40.16	1.41	0.00	13.52	41.94	-3.32
	19.93	4.98	29.41	2.75	0.88	9.57	29.33	-4.19
	20.01	13.02	53.55	0.45	0.00	20.54	57.19	-2.67
	9.99	8.99	36.43	1.82	0.07	11.71	37.68	-3.68
	29.91	8.98	52.51	0.50	0.00	20.00	56.02	-2.69
K_2HPO_4	20.02	9.03	41.68	0.98	0.00	16.47	44.46	-2.69
	20.25	11.00	47.37	0.68	0.00	18.71	50.68	-2.63
	19.31	12.47	50.02	0.57	0.00	19.95	53.64	-2.58
$K_3C_6H_5O_7$	19.93	9.01	29.83	3.20	0.04	20.68	34.53	-1.70
	20.21	11.04	37.69	1.79	0.02	21.72	42.63	-1.89
	19.90	12.66	41.78	1.27	1.27	23.03	47.10	-1.92

Table S5. Phase composition, tie-line data of the ATPS formed by UCON (1) + salt (2)

Salt	overall		Top phase		Bottom phase		TLL	STL
	$100w_1$	$100w_2$	$100w_1$	$100w_2$	$100w_1$	$100w_2$		
$MgSO_4$	20.06	9.02	25.97	3.71	0.06	26.97	34.82	-1.11
	20.17	11.00	29.78	2.18	0.01	29.52	40.43	-1.09
	20.13	12.96	33.28	1.18	0.00	31.00	44.68	-1.12
$Na_3C_6H_5O_7$	19.51	8.62	27.61	4.47	0.43	18.40	30.54	-1.95
	19.93	10.97	33.93	2.83	0.02	22.55	41.35	-1.72
	19.88	12.90	38.22	1.96	0.00	24.75	46.18	-1.68
Na_2SO_4	25.01	9.00	43.88	1.05	0.00	19.54	47.62	-2.37
	14.95	8.99	35.08	1.84	0.15	14.26	37.07	-2.82
	20.02	7.00	34.81	1.87	0.20	13.88	36.63	-2.88
	19.96	10.98	42.68	1.14	0.00	19.62	46.51	-2.31
	20.18	9.04	40.48	1.31	0.01	16.72	43.30	-2.63
	20.04	5.01	25.30	3.23	1.24	11.45	25.43	-2.93
	19.97	12.98	45.18	0.96	0.00	22.51	50.05	-2.10
	9.95	8.95	29.22	2.60	0.89	11.96	29.84	-3.03
	29.97	9.00	47.46	0.82	0.00	24.27	52.94	-2.02
K_2HPO_4	20.71	10.97	40.77	1.10	0.00	21.16	45.33	-2.03
	19.97	9.04	35.19	1.78	0.01	18.56	38.98	-2.10
	19.76	12.87	42.49	0.94	0.00	23.06	47.91	-1.92
$K_3C_6H_5O_7$	19.94	10.96	31.25	3.46	0.00	24.19	37.50	-1.51
	20.23	12.95	35.58	2.61	0.00	26.59	42.90	-1.48
	20.03	15.06	40.30	1.56	0.00	28.10	48.10	-1.53

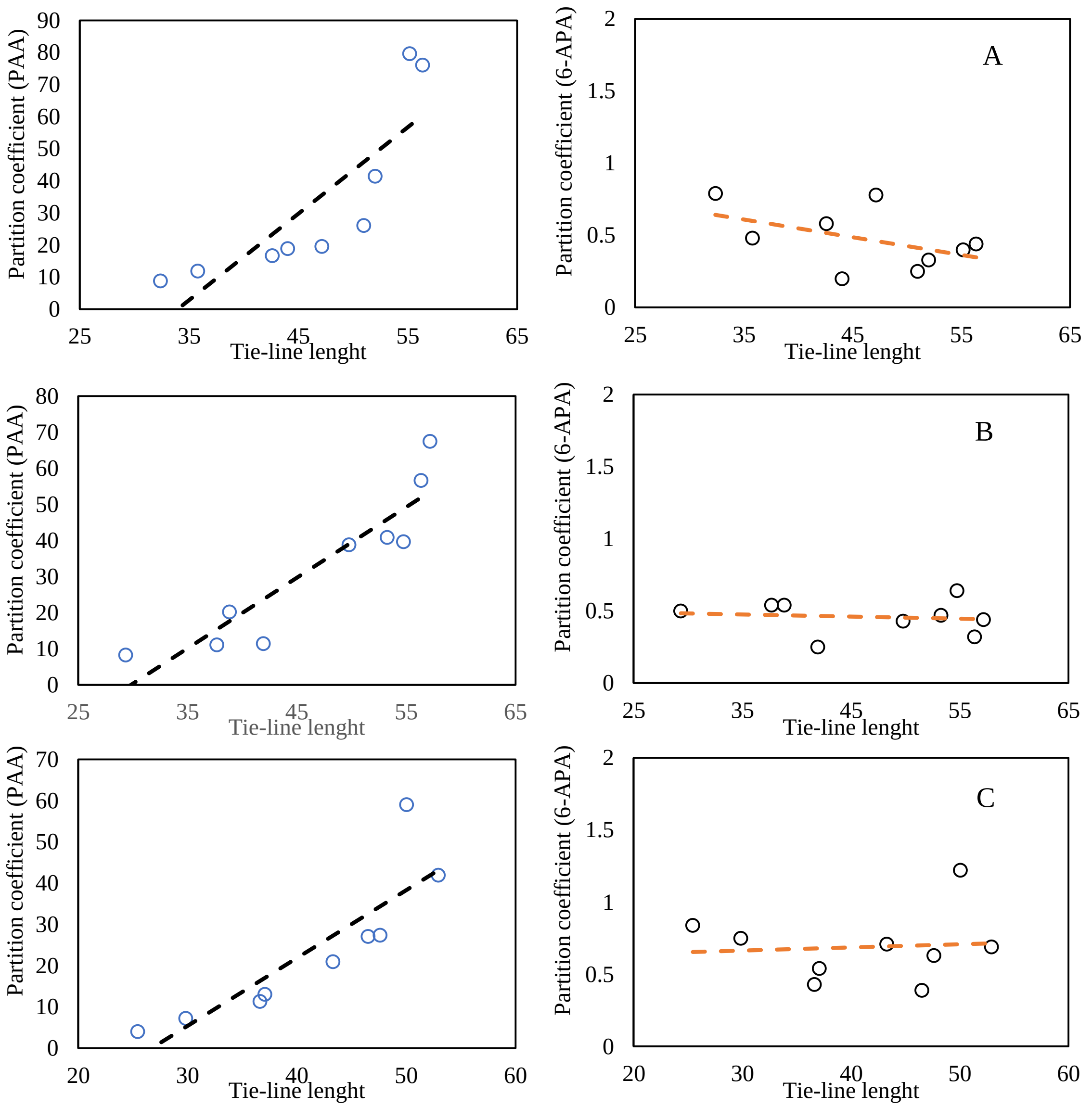

Figure S6. The effect of tie-line length on partition coefficient in ATPSs composed of: A- Pluronic 10R5, B- Pluronic L35 and C- UCON

Table S6. Water ratio between top and bottom phases in copolymer-salt ATPSs

Salt	R-Pluronic 10R5	Pluronic L35	UCON
$MgSO_4$	0.87	0.90	0.96
$Na_3C_6H_5O_7$	0.77	0.75	0.84
Na_2SO_4	0.62	0.52	0.69
K_2HPO_4	0.70	0.69	0.77
$K_3C_6H_5O_7$	0.76	0.85	0.84

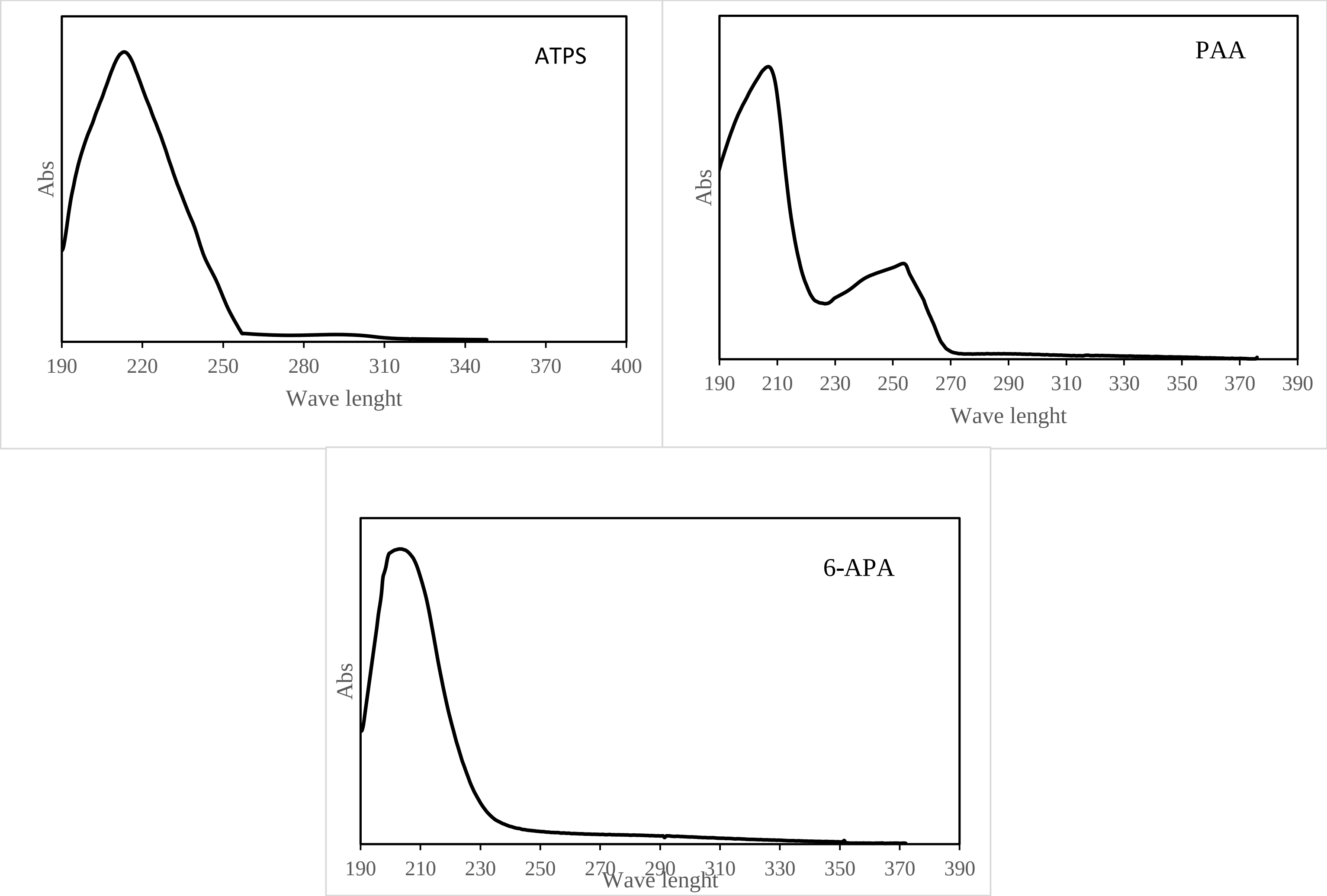

Figure S7. Absorbance spectra
